# Platelet secretions exert anti-inflammatory effects *in vitro* on neutrophils and uterine epithelia in cattle: a possible role in amplifying the uterine immune network toward pregnancy

**DOI:** 10.3389/fimmu.2025.1560996

**Published:** 2025-03-31

**Authors:** Mohamed Samy Yousef, Ihshan Akthar, Dongxue Ma, Shingo Haneda, Kazuya Kusama, Masayuki Shimada, Kazuhiko Imakawa, Akio Miyamoto

**Affiliations:** ^1^ Global Agromedicine Research Center (GAMRC), Obihiro University of Agriculture and Veterinary Medicine, Obihiro, Japan; ^2^ Department of Theriogenology, Faculty of Veterinary Medicine, Assiut University, Assiut, Egypt; ^3^ Department of Clinical Veterinary Medicine, Obihiro University of Agriculture and Veterinary Medicine, Obihiro, Japan; ^4^ Department of Endocrine Pharmacology, Tokyo University of Pharmacy and Life Sciences, Tokyo, Japan; ^5^ Graduate School of Integrated Sciences for Life, Hiroshima University, Higashi-Hiroshima, Japan; ^6^ Research Institute of Agriculture, Tokai University, Kumamoto, Japan

**Keywords:** platelets, neutrophils, endometrium, anti-inflammatory, cattle

## Abstract

**Background:**

Platelet derivatives improve the uterine immune environment and increase pregnancy success in humans and animals. Platelet-conditioned media (PCM) contain all molecules derived from platelets *in vitro* (platelet secretions). The present study aimed to investigate the immunological impacts of platelet secretions on polymorphonuclear neutrophils (PMNs) and bovine endometrial epithelial cells (BEECs), *in vitro*.

**Methods:**

Platelets (10×10^7^ platelets/mL) from Holstein dairy cows were incubated for 0.5 h or lysed to obtain the PCM and platelet lysate (Lysate), respectively. PMNs were stimulated with PCM for 3h. While BEECs were exposed to PCM or Lysate for 24 h. Real-time PCR was performed to detect the expression of targeted genes (cytokines), including *TNFA*, *IL1B* and *PGES1*. Lipoxin A4 (LXA4; anti-inflammatory mediator) and PGE2 concentrations in the supernatants of PMNs cultured with PCM were measured via ELISA. Cell proliferation in BEECs was assessed using the Cell Counting Kit-8 (CCK-8). Additionally, uterine explants were prepared and processed for immunofluorescence to determine the expression of the LXA4 receptor.

**Results:**

In PMNs, platelet secretions downregulated the mRNA expression of pro-inflammatory cytokines (*TNFA* and *IL1B*) and increased LXA4 production. In both PMNs and BEECs, platelet secretions upregulated *PGES1* expression and PGE2 production. In BEECs, platelet secretions and Lysate upregulated *TGFB1.* While Lysate suppressed *IL1B* mRNA expression. Further, platelet secretions showed an anti-proliferative effect in BEECs and increased the LXA4 receptor protein expression in the endometrial epithelia.

**Conclusions:**

Our findings reveal for the first time that platelet secretions directly act on PMNs and BEECs *in vitro*, thereby assisting the uterine immune network to shift anti-inflammatory environment toward pregnancy. The present study can explain, in part, the successful applications of platelet derivatives in reproductive medicine.

## Introduction

Platelets, the smallest non-nucleated blood components, are classified as the major player of primary hemostasis. However, platelets are now considered as an essential regulator of many inflammatory conditions (1). They host a broad spectrum of transcripts, including a variety of messenger RNAs, non-coding RNAs, microRNAs and circular RNAs (2). Their RNAs are complemented by translational machinery, which allows the translation of stored transcripts into proteins after stimulation (3). Moreover, platelets are known to release micro-particles in a regulated manner into the blood or their conditioned media (4, 5). The biogenesis of these particles is determined by various stimuli, leading to differences in their composition and biological roles. The activated platelets by various agonists such as thrombin led to a change in their shape and release of higher quantities of particles (6). The activated particles are mainly protein/lipid-rich, promoting their coagulant activity and inflammatory responses (7). On the other hand, nonactivated platelets release microparticles spontaneously which contribute to the baseline levels of extracellular vesicles in circulation (8, 9). These particles are basically protein-rich, reflecting the resting state of platelets (7). They play a role in maintaining vascular homeostasis and have a potential role in intercellular communication without inducing coagulation (10).

Besides their immune-stimulatory role, platelets also provide an anti-inflammatory impact on different immune cells such as polymorphonuclear neutrophils (PMNs), macrophages and lymphocytes (11–13). It was found that platelets can interact and regulate the function of these cells to produce anti-inflammatory mediators, particularly lipoxin A4 (LXA4) (14). LXA4 is a lipid mediator that elicits anti-inflammatory and pro-resolution actions via its receptor, formyl peptide receptor 2 (LXA4-R) (15). In addition, Prostaglandin E2 (PGE2) and Transforming growth factor beta 1 (TGFB1) are produced by platelets and play a key role in their immune crosstalk with other cells (11, 16). The maternal platelets, due to their small size, are known to be the first among the maternal blood cells that can pass through the narrow intercellular gaps and enter the intervillous space in humans (17). Hence, the interaction between platelets and immune cells is anticipated. Over the last decade, application of platelet rich plasma (PRP) to the uterus for improving pregnancy has gained more attention since they are less invasive, safe and cost-effective (18, 19). PRP can promote the endometrial receptivity and improve pregnancy outcomes in humans and cattle (20, 21). Moreover, PRP has been shown to enhance embryo recovery in mares, particularly those susceptible to persistent breeding-induced endometritis (PBIE). While, platelet-poor plasma (PPP) does not exhibit the same efficacy (22). This comparison highlights the importance of platelet-derived factors in these processes and shows that the other plasma components within the PPP do not produce the same result. Further, PRP also known to reduce the inflammation in bovine endometrial cells, as evidenced by downregulation of pro-inflammatory genes expression after stimulation with bacterial endotoxins (20). Moreover, intrauterine infusion of autologous PRP improved conception rates in repeat breeder cows (23). In addition, platelet lysate (Lysate) reduces the inflammatory response of the equine uterus when administered pre- or post-artificial insemination (AI) (24). However, the mechanism underlying the immune modulatory effects of platelets and their derivatives remains unclear.

The influence of platelets and their derivatives on other cells has been investigated using different experimental approaches. For instance, the impact of PRP was evaluated in various species using different biological models including reproductive immunology (21, 25). PRP intrauterine infusion reduced inflammation and apoptosis of the endometrial cells after Lipopolysaccharides (LPS) treatment using a mouse model (26). Notably, bovine uterine immunity is characterized by a delicate balance between immune tolerance, necessary for successful pregnancy, and vigorous defense mechanisms against prevalent uterine diseases. These diseases, including subclinical and clinical endometritis, and metritis, are major causes of reproductive failure in cattle. Unlike humans, where decidualization plays a dominant role in early pregnancy, bovine uterine immunity relies heavily on innate immune responses, particularly neutrophil function, to manage bacterial contamination post-partum (27). The high prevalence of bacterial infections in the bovine uterus contributes to the significant economic losses associated with these diseases. Given the increasing concern of antimicrobial resistance, alternative therapeutic strategies are crucial (28). PRP and platelet lysate, offer promising avenues for modulating uterine inflammation and improving conception rates in cattle (20, 23). However, it is noteworthy that the plasma contains various bioactive compounds other than platelets. For instance, small Extracellular Vesicles (sEVs) play a significant role in neutrophil activity and uterine tissue dynamics. Studies have shown that sEVs derived from high-fertile dairy cow plasma can downregulate pro-inflammatory cytokines in bovine endometrial epithelial and stromal cells, indicating a potential role in mitigating neutrophil-mediated inflammation (29). sEVs carry various bioactive molecules, including microRNAs, proteins, and lipids, that can modulate cellular signaling and function. Hence, the present study was designed to explore the immunological impacts of platelet secretions (without plasma) on neutrophils and uterine epithelial cells in cattle. By elucidating the immunomodulatory mechanisms of these platelet-derived factors, we aim to provide a foundation for developing novel therapeutic approaches to enhance bovine reproductive health.

## Methods

### Ethics statement

The protocol was approved by the Committee on the Ethics of Animal Experiments of the Obihiro University of Agriculture and Veterinary Medicine, Japan (Permit number 27-74).

### Experimental design

**Figure 1:**
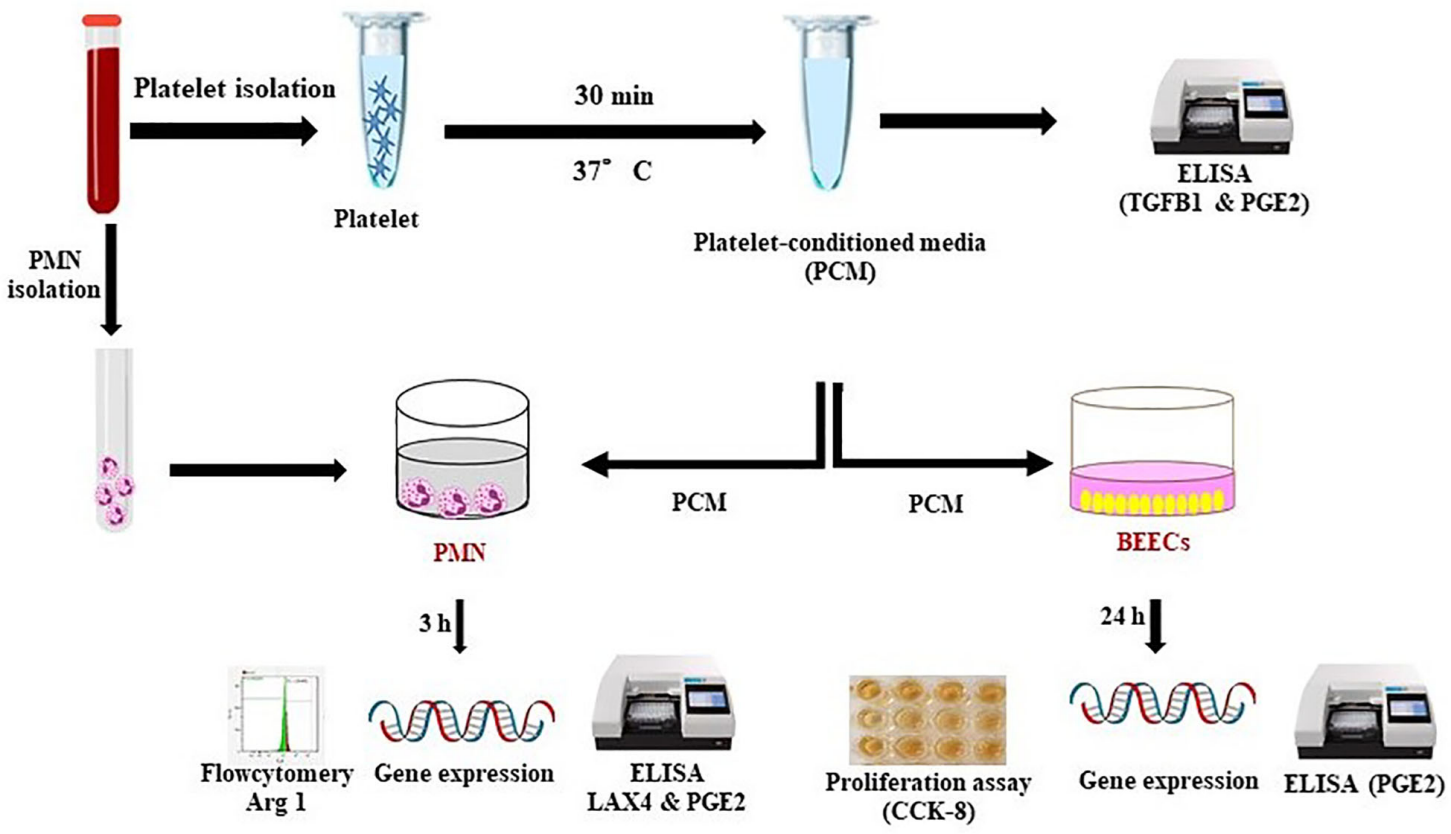
Schematic representation of the experimental design. For investigating the platelets’ communication with PMN and bovine uterus via their secretion, PMN or BEECs were exposed to the PCM (collected after 0.5 h incubation). Later on, cells were analyzed for gene expression by RT-PCR. Besides, PMNs phenotyping was examined by flow cytometry using Arg 1 (anti-inflammatory intracellular marker). Using commercial ELISA kit, the *TGFB1* concentration was quantified to examine the platelets’ functionality. Moreover, ELISA was also performed to determine the PGE2 levels in conditioned media after different treatments.

For investigating the platelets’ communication with PMN and bovine uterus via their secretion, PMN or bovine endometrial epithelial cells (BEECs) were exposed to the platelet conditioned media (PCM). Cells were analyzed for gene expression by real-time PCR. Besides, PMNs phenotyping was examined by flow cytometry using arginase 1 (Arg 1) (anti-inflammatory intracellular marker). Using ELISA, the *TGFB1* concentration was quantified to examine the platelets’ functionality and further PGE2 levels were determined in the conditioned media after different treatments (**Figure 1**).

### Blood platelets’ isolation and preparation of PCM and Lysate

Blood was collected from the tail veins of multiparous Holstein dairy cows (n=3) at the Obihiro University of Agriculture and Veterinary Medicine (OUAVM) farm. The platelets were isolated and the PCM were prepared as previously described (13). Platelets were incubated at 37°C for 0.5 h and then centrifuged at 2500 ×g for 5 min. Then, the supernatant (PCM) was collected and stored at -30°C till using in experiments as described below. Lysate was prepared as previously described by Strandberg et al. (30) with minor modification. In brief, platelets (10×10^7^/mL of Tyrode’s buffer) were lysed by freezing at -80°C for at least 10 min and thawing in a water bath at 37°C for 7.5 min. Then, supernatants (Lysate) were collected after centrifugation at 2500 ×g for 5 min at 25°C after 3 freeze-thaw cycles. Supernatants were aliquoted and stored at -80°C till use.

### Detection of TGFB1 level in PCM and Lysate

The concentration of *TGFB1* in PCM and Lysate was measured using a bovine TGFB1 ELISA kit (Cat No. MBS2886167, MyBioSource.com) according to the manufacturer’s instructions. The kit has a sensitivity with the minimum detection limit of 19.5 pg/mL as well as a wide quantification range of 78-5000 pg/mL. All samples were run in duplicate. Optical density (OD) readings were performed at 450 nm.

### Preparation and stimulation of PMNs

PMNs were isolated as described previously (31). Basically, blood collection experiments were conducted at the Field Center of Animal Science and Agriculture of Obihiro University, Heparinized blood from a multiparous Holstein cow in luteal stage was collected and mixed with an equal volume of PBS^−/−^, slowly layered over Ficoll-Paque solution (Lymphoprep, Axis Shield, Oslo, Norway), and centrifuged at 1000 g for 30 min at 10°C. PMN layer was mixed with ammonium chloride lysis buffer (NH4Cl, 155 mM; KHCO3, 3.4 mM; and EDTA, 96.7 μM) for 10 s and then centrifuged at 500 g for 10 min at 10 °C to purify PMNs from red blood cells. After centrifugation, the cell pellet was washed two times with PBS^−/−^. The purity of PMNs was evaluated by flow cytometry was >98%, and the viability was around 99% as assessed by Trypan Blue staining. PMNs were suspended at a density of 1×10^7^ cells/mL with PCM in a culture tube and incubated for 3 h with gentle shaking. After incubation, PMNs were centrifuged, lysed in Trizol, and stored at -80°C until RNA extraction. While the supernatant was collected and stored in aliquots at -80°C till further use.

### Preparation of BEECs and stimulation with PCM and Lysate

BEECs were isolated and then cultured according to the previously described protocols (32, 33). In brief, uterine samples from cows were collected at a local slaughterhouse (Hokkaido Livestock, Doto plant, Obihiro, Japan). Uteri from late estrous cycle (diestrus) cows (Days 2–5) were used. A polyvinyl catheter was inserted into the oviduct, and the horn near the corpus uteri was tied to retain the collagenase solution for epithelial cell solubilization, as described below. The uterine lumen was washed three times with 30–50 ml of sterile Hanks’ balanced salt solution (HBSS), supplemented with 100 IU/ml penicillin, 100 μg/ml streptomycin, and 0.1% BSA (Boehringer Mannheim GmbH, Mannheim, Germany; #735078). Subsequently, 30–50 ml of enzyme solution (sterile HBSS containing 0.05% collagenase I [Sigma Chemical Co., St. Louis, MO; #C-0130], 0.005% deoxyribonuclease I [Sigma; #D-5025], and 0.1% BSA) was infused into the uterine lumen through the catheter. Epithelial cells were isolated by incubating the tissue at 37°C for 30 minutes, with gentle shaking. The cell suspension from the digestion was filtered through metal mesh (100 μm) to remove undissociated tissue fragments. The filtrate was washed three times by centrifugation (10 minutes at 100 × g) with Dulbecco’s modified Eagle’s medium (DMEM; Sigma; #D-1152), supplemented with antibiotics and 0.1% BSA. After washing, cells were counted using a hemocytometer, and cell viability was assessed by 0.5% trypan blue dye exclusion, with viability exceeding 95%. The purity of epithelial cells in our model (>98%) was ensured as described previously (34). Sub-confluent BEECs monolayers in 24-well plates were incubated in 1 mL/well with PCM or Lysate for 24 h. BEECs incubated in Tyrode’s buffer were kept as a control group. This experiment was repeated four times using epithelial cells from four different uteri.

### Quantitative real-time PCR

RNA extraction, cDNA synthesis, and quantitative real-time PCR were performed following the protocol described previously (35). Quantitative real-time PCR of targeted genes (**Table 1**) was performed using SYBR Green PCR Master Mix (Bio-Rad Laboratories) by using CFX Connect™ Real Time PCR detection system (Bio-Rad Laboratories). The amplification program was set up according to the previous protocol (35). The calculated cycle threshold values were normalized using *β-actin* as the internal reference gene by applying the Delta-Delta comparative threshold method to quantify the fold change between samples.

**Table 1:** List of primers used in real-time PCR.

Gene	Primer	Sequence of nucleotide (5′⇒3′)	Accession No.
β-actin	F R	TCACCAACTGGGACGACATG CGTTGTAGAAGGTGTGGTGCC	AY141970.1
*TGFB1*	F R	CTTTCTTCAAATGCAGCATTGG GGGTCTGGGTGATACAACGAA	NM_001166068.1
*TNFA*	F R	CAAAAGCATGATCCGGGATG TTCTCGGAGAGCACCTCCTC	NM_173966.3
*IL1B*	F R	AATCGAAGAAAGGCCCGTCT ATATCCTGGCCACCTCGAAA	NM_174093.1
*PGES1*	F R	AAAATGTACGTGGTGGCCGT CTTCTTCCGCAGCCTCACTT	NM_174443.2

### Flow cytometry

Representative phenotypic marker for anti-inflammatory N2 PMN (arginase 1; Arg 1) was determined using polyclonal antibody according to the manufacturer’s protocol. In brief, PMNs (1x10^6^) were fixed, permeabilized with 0.2% Triton-X (10 min) and cells were blocked in 5% BSA for 1 h at room temperature, followed by incubation with Anti-Liver Arginase antibody (1:100, Abcam, ab96183) overnight at 4°C. After washing three times with PBS, the cells were incubated with Alexa Flour conjugated secondary antibody (1:200, Invitrogen, A-11035) for 1h at 4°C. Data was acquired using SA3800 Spectral Analyzer (Sony Biotechnology, Tokyo, Japan).

### Immunofluorescence of uterine explants

Uterine explants were prepared and processed for immunofluorescence as described previously (36). In brief, uterine samples of cows were collected from the local slaughterhouse (Hokkaido Livestock, Doto plant Tokachi Factory, Obihiro, Hokkaido, Japan). The reproductive tracts were trimmed free of surrounding tissues, opened and macroscopically examined to be free from pus, inflammation, and abnormal color. The phase of the estrous cycle was identified based on the appearance, weight, and color of the corpus luteum and follicular diameter. The uteri from the late estrous cycle (diestrus) cows (Days 2–5) were used. The horn ipsilateral to the corpus luteum was transported on ice to the lab and longitudinally incised. Endometrial tissue samples were taken from the intercaruncular regions using an 8 mm biopsy punch. The functional endometrial layer was isolated with surgical scissors. Explants were placed epithelial side up in TALP medium, maintained at 38.5°C, and incubated at 38.5°C with 5% CO2 for 15 minutes. Tissue integrity was confirmed by histology using hematoxylin and eosin and minimal apoptosis was verified by analyzing Caspase 3 mRNA expression. Sections were incubated with primary antibodies for LXA4-R (FPR2- specific rabbit polyclonal, 1:200, cat. no. 720293, Thermo Fisher Scientific) at 40°C in a humidified chamber overnight. The sections were then labeled with Alexa Fluor goat anti-rabbit secondary antibodies (1:200, A-11035 or A11034, Thermo Fisher Scientific) for 30 min. Sections were washed, and coverslips were mounted using VECTASHIELD mounting medium containing DAPI (H-1000; Vector Laboratories). The fluorescence signal was then captured using an all-in-one fluorescence microscope (Keyence, BZ-X800) using the BZ-X GFP, BZ-X Texas Red and BZ-X DAPI filters. Exposure time was kept constant for the primary antibody and its negative control.

### Proliferation assay

Cell proliferation rates were measured using the Cell Counting Kit-8 (CCK-8) assay (CK04; Dojindo Molecular Technologies, Inc., Kumamoto, Japan). Briefly, the epithelial cells were detached and suspended in Dulbecco’s Modified Eagle Medium: Nutrient Mixture F12 (DMEM/F12) (Gibco) supplemented with 22 mM NaHCO3 (Sigma-Aldrich, St. Louis, MO, USA), 0.1% gentamicin (Sigma-Aldrich), 1% amphotericin B (Gibco), and 10% heat-inactivated fetal calf serum (FCS) (Bio Whittaker, Walkersville, MD, USA). Then, cells were seeded in 25 cm2 culture flasks (Nalge Nunc International, Roskilde, Denmark), and cultured at 38.5˚C in a humidified atmosphere of 5% CO2 in air. Upon reaching 70-80% (sub-confluence). BEECs, following first passage, were trypsinized and seeded in 1.5 ml/well culture medium (DMEM/F12, 22 mM NaHCO3, 0.1% gentamicin, 1% amphotericin, and 5% FCS) onto 96-well culture plates pre-coated with type I collagen (1 × 10^4^ cells/well). BEECs were co-cultured with PCM or Lysate for 24 h. Cell proliferation rates were measured for 24 h using the Cell Counting Kit-8 (CCK-8) assay (CK04; Dojindo Molecular Technologies, Inc., Kumamoto, Japan). A total of 10 μL of CCK-8 solution (Dojindo, Tokyo, Honshu, Japan) was added to each well at 0 h and 24 h. The cells cultured with vehicle/Tyrode’s buffer were kept as a control group. The absorbance at 450 nm was read using a microplate reader (Labsystem Multiskan MS 352, Labsystems, Finland) after 2 h incubation. The current assay was performed in triplicate.

### TUNEL assay

After coculture of BEECs with PCM or Lysate for 24h, apoptotic cell death was evaluated by TUNEL assay using the *In situ* cell death detection kit, Fluorescein (catalog no. 11684795910; Roche Diagnostics GmbH, Mannheim, Germany) according to the manufacturer’s protocol. Next, the cells were mounted in VECTASHIELD mounting medium containing DAPI (H-1200, Vector Laboratories, CA, USA). The apoptotic cells were observed under a fluorescence microscope (BZ-X800).

### Determination of LXA4 and PGE2 concentrations

LXA4 concentrations in the supernatants of PMNs that cultured in the presence of PCM were measured by commercially available ELISA kit (Neogen, Lexington, KY; range was 20 to 2000 pg/mL). The assay was performed following the instructions of the manufacturer and measured by a microplate reader at 650 nm. Moreover, the concentration of PGE2 in the culture media was determined using a Prostaglandin E2 ELISA Kit (514010, Cayman Chemical Co., Ann Arbor, MI) in accordance with the manufacturer’s instructions. The absolute production of PGE2 by PMNs and BEECs was calculated after subtracting out the amounts of PGE2 that were detected in the PCM or Lysate.

### Statistical analysis

The statistical analysis was conducted with SPSS^®^ software version 22 (IBM, Armonk, Ny, USA). The data were first tested for normality using Kolmogorov–Smirnov test. Statistical differences were determined using a Student’s *t*-test (for two groups; PMNs’ data) or One-Way ANOVA followed by Bonferroni’s *post hoc* test (for more than two groups; BEECs’ data). The results were presented as mean ± standard error of the mean (SEM). The statistical significance was set at *p* ≤ 0.05.

## Result

### PGE2 and TGFB1 concentrations in PCM and Lysate

**Figure 2:**
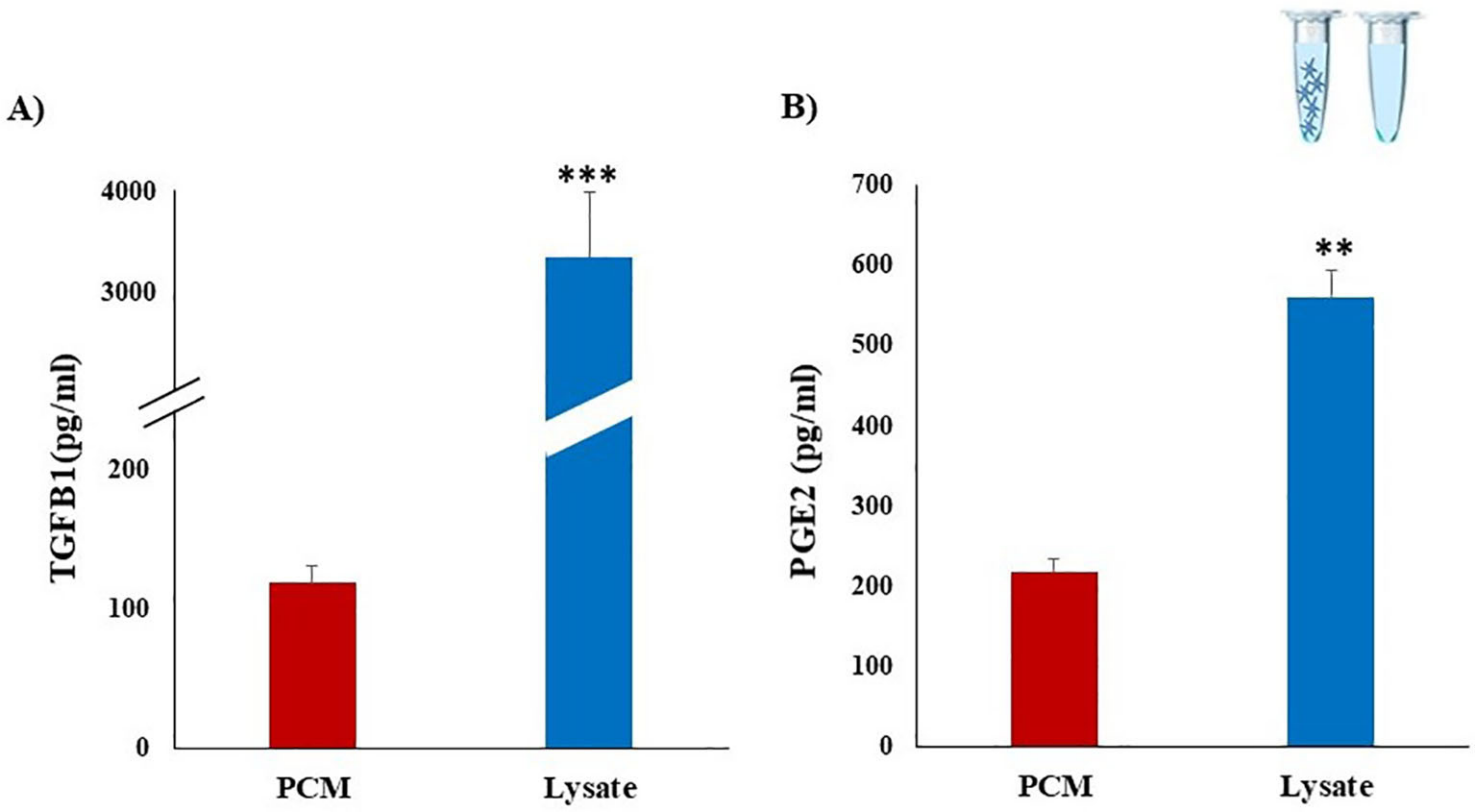
Detection of TGFB1 and PGE2 concentrations in platelet derived media using ELISA assays. Platelets were isolated from whole blood collected from three multiparous Holstein dairy cows (n=3). PCM were obtained after culturing of platelets (10 x 10^7^) for 30 min. Platelet lysate (Lysate) was prepared by repeated freezing and thawing cycles of platelet concentrates. Considerable concentrations of TGFB1 **(A)** and PGE2 **(B)** were detected in PCM and Lysate. Dilution buffer (Tyrode’s buffer) served as the negative control media. All samples were run in duplicate. Data represent means ± SEM; asterisks denote statistical differences: **p < 0.01 and ***p < 0.001 determined by a student’s t-test.

We investigated whether bovine platelets could produce and store TGFB1 and PGE2, by using ELISA. Results showed that PCM contains a considerable concentration of PGE2 and TGFB1 (234.9 ± 38.3 and 118.7 ± 14.9 pg/mL, respectively). While the highest levels of TGFB1 (3678.3 ± 467.1 pg/mL) and PGE2 (560.4 ± 32.2 pg/mL) were quantified in the Lysate group (**Figure 2**). The higher levels of TGFB1 and PGE2 in the Lysate group likely reflect the broader and more comprehensive exposure to platelet-derived bioactive molecules compared to the more selective release observed in the PCM group.

### PCM upregulated anti-inflammatory response in PMNs

**Figure 3:**
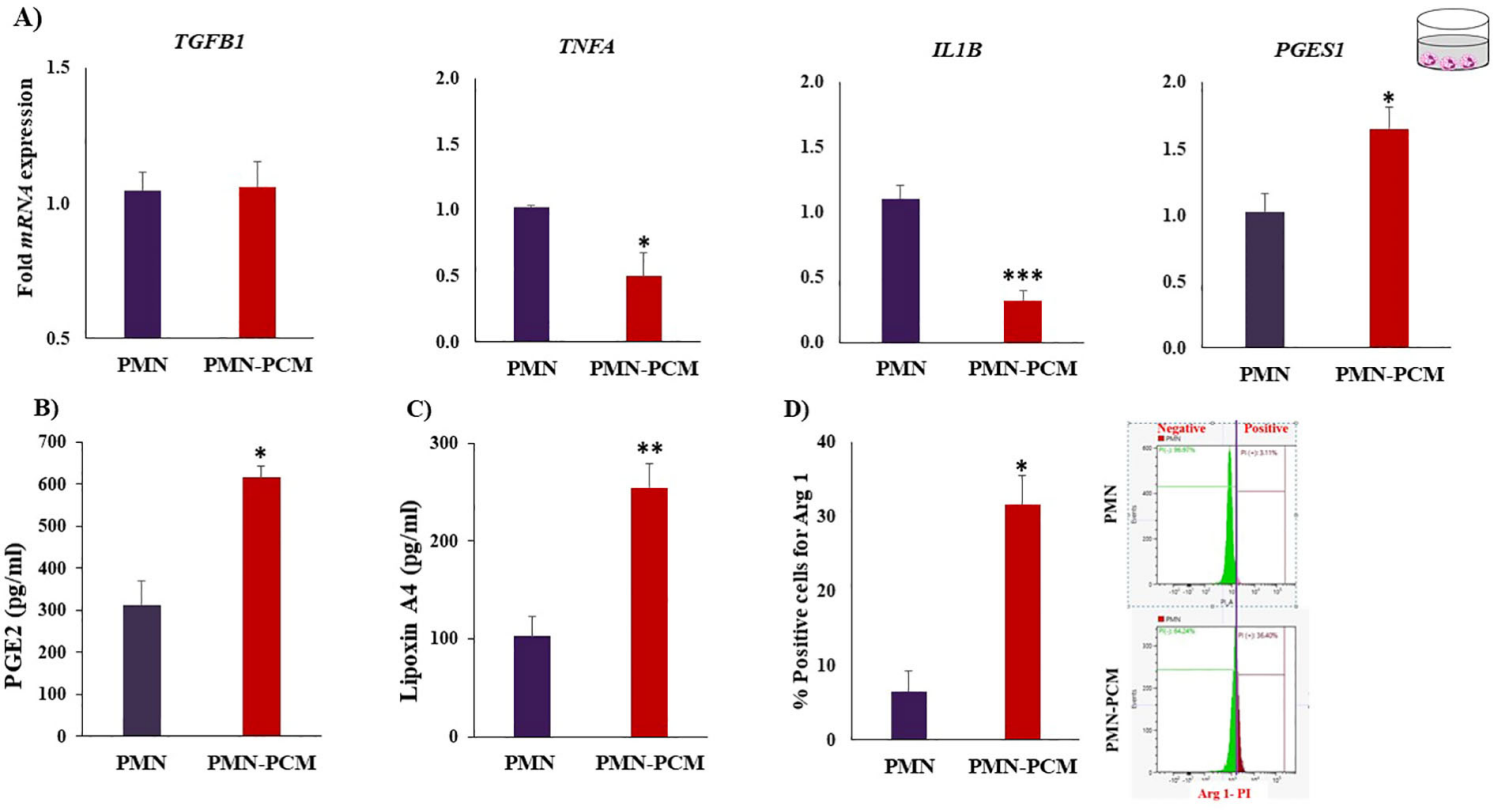
PCM shift the PMNs toward anti-inflammatory response. PMNs were isolated and cultured with PCM for 3h. **(A)** PCM suppressed the mRNA expression of pro-inflammatory cytokines (*TNFA* and *IL1B*) and induced the expression of *PGES1* quantified by RT-PCR (n = 4). **(B, C)** Determination of PGE2 and LXA4 concentrations produced by PMNs after incubation with PCM using ELISA. PCM contain no or below detectable levels of the LXA4. **(D)** PCM induced arginase-1 expression in PMNs. PMNs were cultured with PCM for 3h. Then, PMNs phenotyping was examined by flow cytometry using Arg 1 (anti-inflammatory intracellular marker). Each experiment was repeated in triplicate. Data are presented as the mean ± SEM. Asterisks denote statistical differences: *p < 0.05, **p < 0.01 and ***p < 0.001 determined by a student’s t-test.

PCM suppressed (*p* < 0.05) the mRNA expression of pro-inflammatory cytokines (Tumor necrosis factor-alpha *(TNFA*) and Interleukin-1beta *(IL1B*)) and increased the expression of *PGES1* (**Figure 3A**). Furthermore, we measured the production of PGE2 (**Figure 3B**) and LXA4 (**Figure 3C**) by PMNs after exposure to PCM. ELISA of the present study showed that PCM could increase (*p* ≤ 0.05) the PGE2 and LXA4 release from PMNs. Of note, PMNs alone produced detectable amounts of LXA4. Importantly, the flow cytometry analysis showed that PCM induced Arg 1 protein expression in PMNs (**Figure 3D**).

### PCM and Lysate upregulated anti-inflammatory response in BEECs

**Figure 4:**
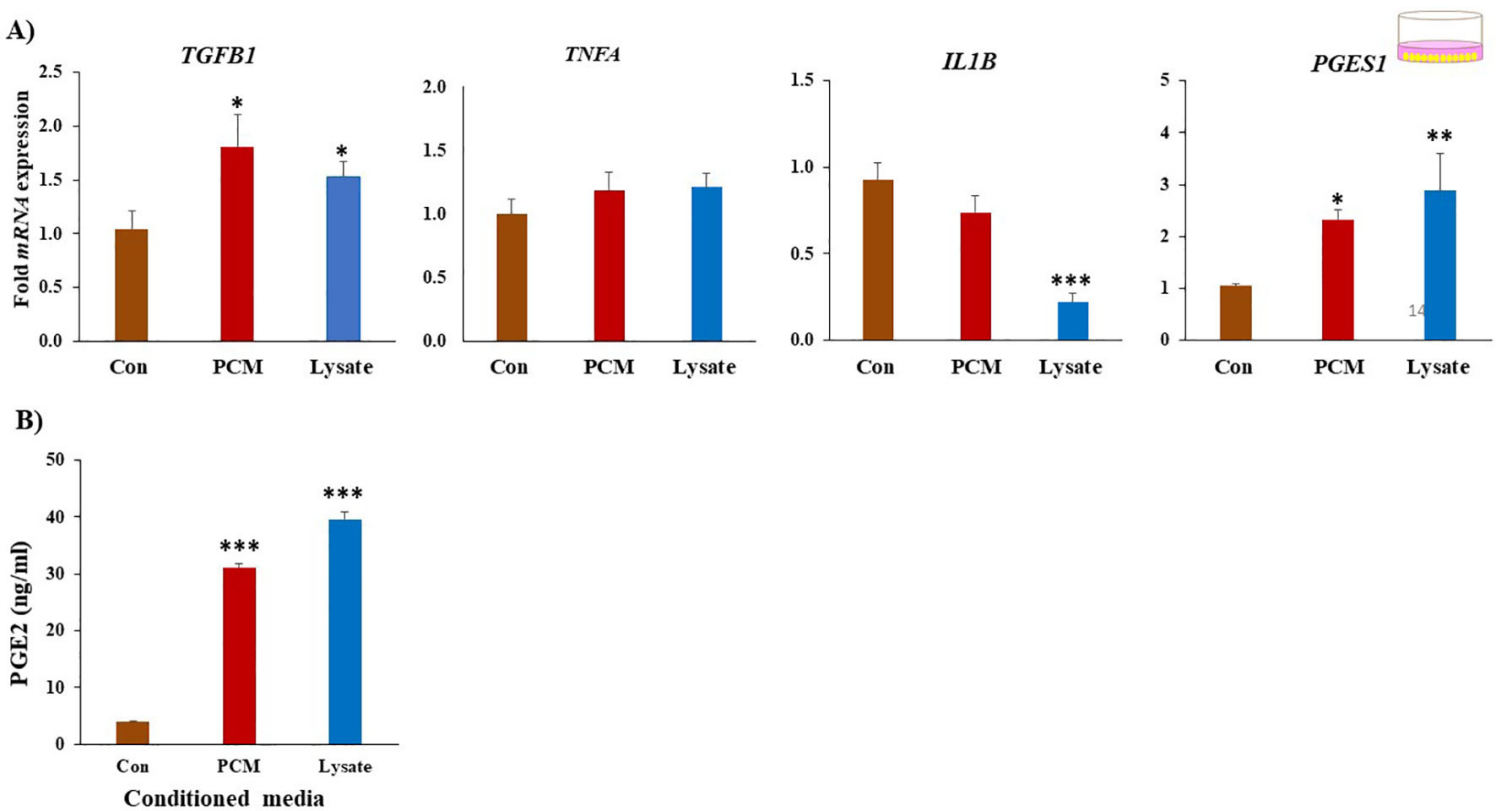
PCM have anti-inflammatory on BEECs. **(A)** Effect of PCM and Lysate on the gene expression of BEECs. BEECs were exposed to PCM or lysate for 24 (h) The mRNA expression of multiple genes of interest was quantified by RT-PCR. Both PCM and Lysate upregulated the *TGFB1*, and Lysate suppresses *IL1B* in BEECs. **(B)** Both PCM and Lysate increased PGES1 expression and PGE2 production in BEECs comparing to control (Con). Results are presented as the mean ± SEM of four independent experiments performed in triplicates. Different asterisks/letter denote statistical differences: *p < 0.05, **p < 0.01 and ***p < 0.001 when compared to the control using a One-Way ANOVA followed by Bonferroni’s test.

**Figure 5:**
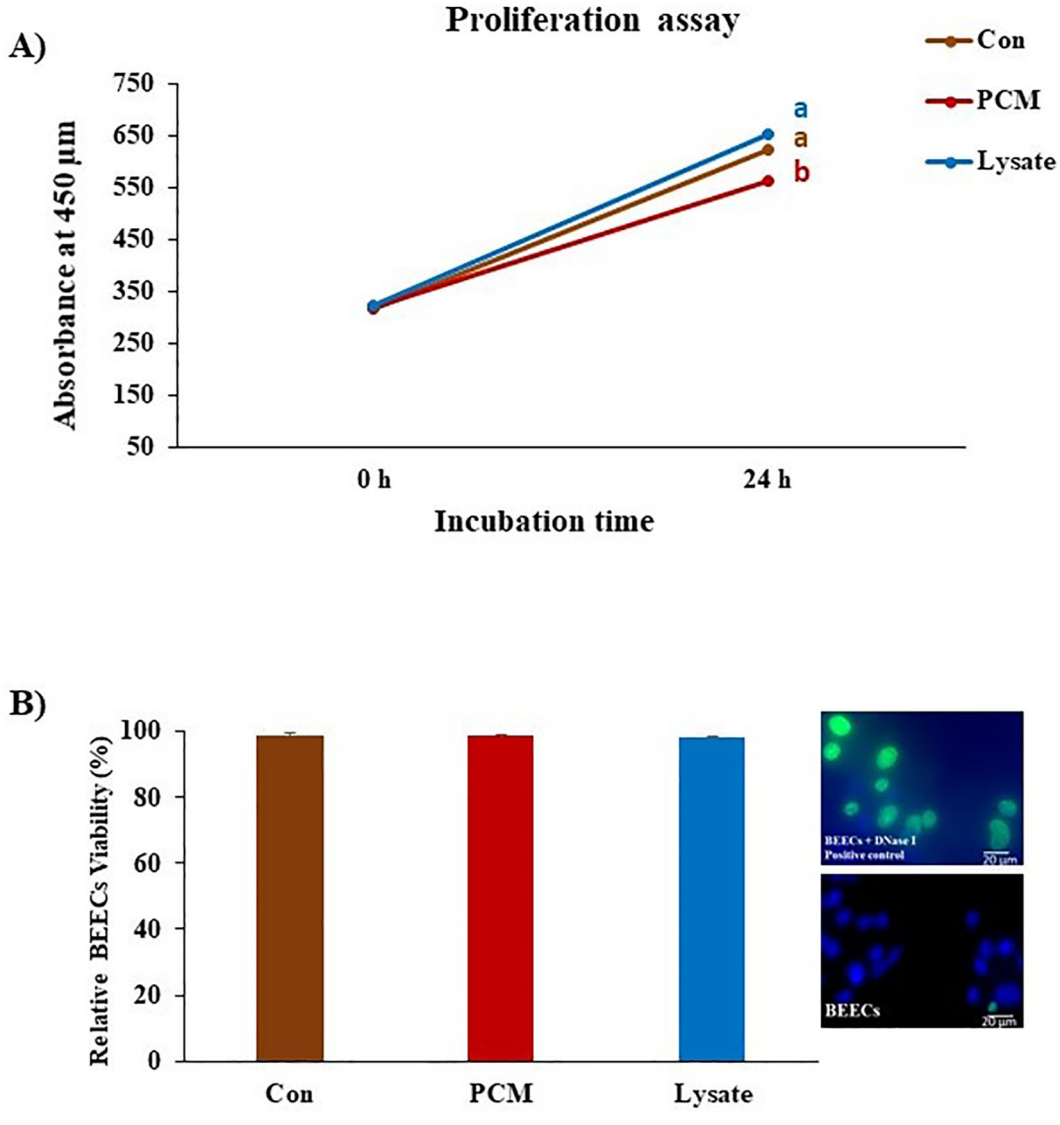
Effect of PCM and Lysate on proliferation and apoptotic status of BEECs. BEECs monolayers were exposed to PCM and Lysate for 24 h followed by a CCK-8 and TUNEL assays to evaluate the proliferation and apoptotic status of BEECs, respectively. **(A)** CCK-8 analysis: PCM showed anti-proliferative effect on BEECs. The values of CCK-8 were measured at 450 nm at 0 and 24 h with a microplate reader. **(B)** TUNEL assay: there was no visible effect of different treatments on the BEECs viability. Results are presented as the mean ± SEM of three independent experiments. Different letters denote a significant variance (p < 0.05) between the different groups using One-Way ANOVA followed by Bonferroni’s *post hoc* test.

Both PCM and Lysate upregulated (*p* < 0.05) the *TGFB1* and *PGES1* mRNA expression in BEECs. Lysate suppressed (*p* < 0.001) *IL1B* mRNA expression (**Figure 4A**). Notably, both PCM and Lysate increased (*p* < 0.05) the production of PGE2 in BEECs (**Figure 4B**). Moreover, BEECs proliferation was inhibited by exposing BEECs to PCM but not with Lysate (**Figure 5A**). Further, PCM and Lysate have no adverse impact on the BEECs viability as detected by TUNEL assay (**Figure 5B**).

### PCM increased the expression of LXA4-R protein in bovine endometrium

**Figure 6:**
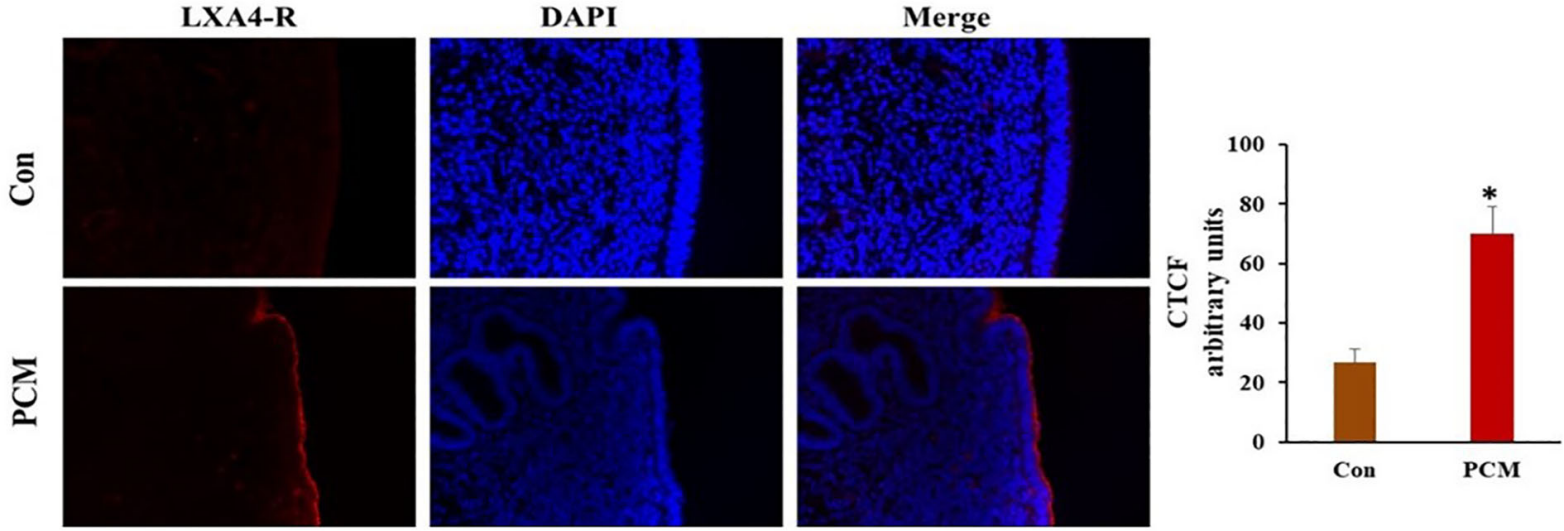
Immunofluorescence localization of Lipoxin A4 receptor (LXA4-R) in bovine endometrium. Uterine explants were incubated in the presence of PCM or without treatment (Con) for 4 h. The localization was observed by immunofluorescence labeling with the Alexa-Fluor-conjugated anti- LXA4-R antibody. The expression was increased in the treated explants especially in the luminal epithelium. Bar = 20µm. The y-axis represents CTCF (corrected total cell fluorescence) staining intensity measured in arbitrary units, which were quantified based on the immunohistochemical staining of the tissue samples. Asterisk denote statistical differences: *p < 0.05, determined by a student’s t-test.

The protein expression of LXA4 receptor (LXA4-R) was increased in the luminal epithelium of the uterine explants exposed to PCM (**Figure 6**).

## Discussion

Since platelets are known to improve fertility and recognized as critical effector cells in different immune responses, we investigated its role in immune response in the bovine uterus. The present data reported the ability of platelets to communicate with PMNs and endometrial epithelial cells via their secretions. Shifting of the immune environment to anti-inflammatory and the regulation of the proliferative status of the uterus by platelet secretions explain the promising use of platelets and its derivatives to improve the reproductive performance in cattle.

In the present study, we reported the ability of bovine platelets to produce and store high levels of *TGFB1* and PGE2. Notably, the obtained findings showed that platelet secretions could modulate PMN’s gene expression toward the anti-inflammatory neutrophil phenotype (N2) *in vitro*. Further, platelet secretions suppressed the mRNA expression of pro-inflammatory cytokines (*TNFA and IL1B*) and increased the protein expressions of Arg 1 (N2 phenotypic marker) in PMNs. The observed basal cytokine levels expressed by epithelial and immune cells are likely a consequence of the *in vitro* handling as reported before (35, 37, 38), while the reduction upon PCM or lysate exposure reflects the inherent anti-inflammatory properties of platelet-derived factors. These anti-inflammatory effects may be mediated, at least in part, by PGE2 derived by platelets as well as the stimulation of PGE2 and LXA4 production from PMNs after incubation with the platelet secretions. Although PGE2 is generally considered to be a proinflammatory molecule (39), our earlier findings and other recent studies reported the basic role of PGE2 to generate the anti-inflammatory response in PMNs and other cells (35, 40, 41). Our data agreed with the previous study in that platelet secretions induce M2 anti-inflammatory macrophages phenotype *in vitro* which is mediated by PGE2 (13). Similarly, the previous reports in mice showed that platelet secretions reduce TNFA production (11, 42) and increase the arginase expression in macrophage (43).

Interestingly, the results of our investigation showed that platelet secretions could induce LXA4 production (anti-inflammatory mediator) from PMNs. As seen in humans, we found that the bovine PMNs alone showed the ability to produce LXA4 to the detectable level. Of note, the direct interactions between platelets and PMNs lead to the transcellular metabolism of LXA4 as a main resolving factor (44). However, platelet secretions contain the platelet-derived microparticles that can transfer a special enzyme (12-LO) to PMNs as an alternative pathway for LXA4 biosynthesis (45). It is well established that LXA4, through its receptor LXA4-R, can modulate PGE2 production. LXA4 has been shown to inhibit the production of pro-inflammatory mediators, including PGE2, in various models of inflammation (46, 47). This suggests that LXA4-R activation can lead to downstream signaling events that suppress PGE2 synthesis. While the exact molecular mechanism of this inhibition is not fully understood, a recent study indicated that both PGE2 and LXA4 values were significantly lower in neuroinflammation patients, suggesting a potential link in their roles to regulate inflammation (48). Additionally, PGE2 is known to stimulate the production of LXA4 in neutrophils to initiate the resolution of inflammation (40, 49).

In BEECs, the results showed that platelet secretions induced an anti-inflammatory effect. It should be noted that LXA4-R was detected and upregulated by platelet secretions in the uterine epithelia. It was found that LXA4-R expression was elevated in the human endometrium during early pregnancy (15). This may be due to the local production of LXA4 in the endometrium, or other anti-inflammatory ligands that can activate LXA4-R (i.e., annexin A1) (15, 50), thereby ensuring the local immune environment toward Th2. Previous studies have shown that LXA4-R activation can influence TGFB signaling. For example, research indicates that LXA4-R activation reduces TGFB levels and exhibits anti-fibrotic effects (51). This suggests that LXA4-R signaling can modulate the downstream effects of TGFB, potentially by interfering with TGFB receptor signaling or by regulating the expression of TGFB target genes. Additionally, studies in osteoarthritis models demonstrate a modulation of TGFB by LXA4, that plays a role in resolving inflammation (52). Further investigation is needed to determine the precise molecular mechanisms by which LXA4-R signaling interacts with TGFB signaling pathways. Thus, the bovine uterus could be responsive also to LXA4 and work as one of the systems for setting an anti-inflammatory environment *via* interactions with other pathways (e.g., TGFB).

In the present study, platelet secretions show anti-proliferative effect in BEECs. A similar trend was observed previously (53) where the human platelet lysate weakened osteogenic cell proliferation *in vitro*. This anti-proliferative action of platelet secretions is likely to contribute to maintain the stable endometrial condition that is a prerequisite for embryo implantation. In response to the pregnancy hormone (progesterone; P4), epithelial cells exit from the cell cycle, maintain the non-proliferative state, and enter a differentiation pathway to acquire the receptive state that supports embryo implantation (54). Moreover, PRP inhibits apoptosis and induces the proliferation of endometrial stromal cells in human (55). The cellular mechanisms by which platelet secretions induce anti-proliferative effects on BEECs are still unknown.

While this study examined the effects of unfractionated platelet secretions, specifically PCM and Lysate, it is known that these preparations contain a complex mixture of bioactive molecules such as cytokines and microRNAs. Future studies employing fractionation techniques for bovine platelets, such as targeted proteomics, are warranted to isolate and identify the specific platelet-derived factors that contribute to the observed anti-inflammatory effects on neutrophils and uterine epithelia in cattle.

**Figure 7:**
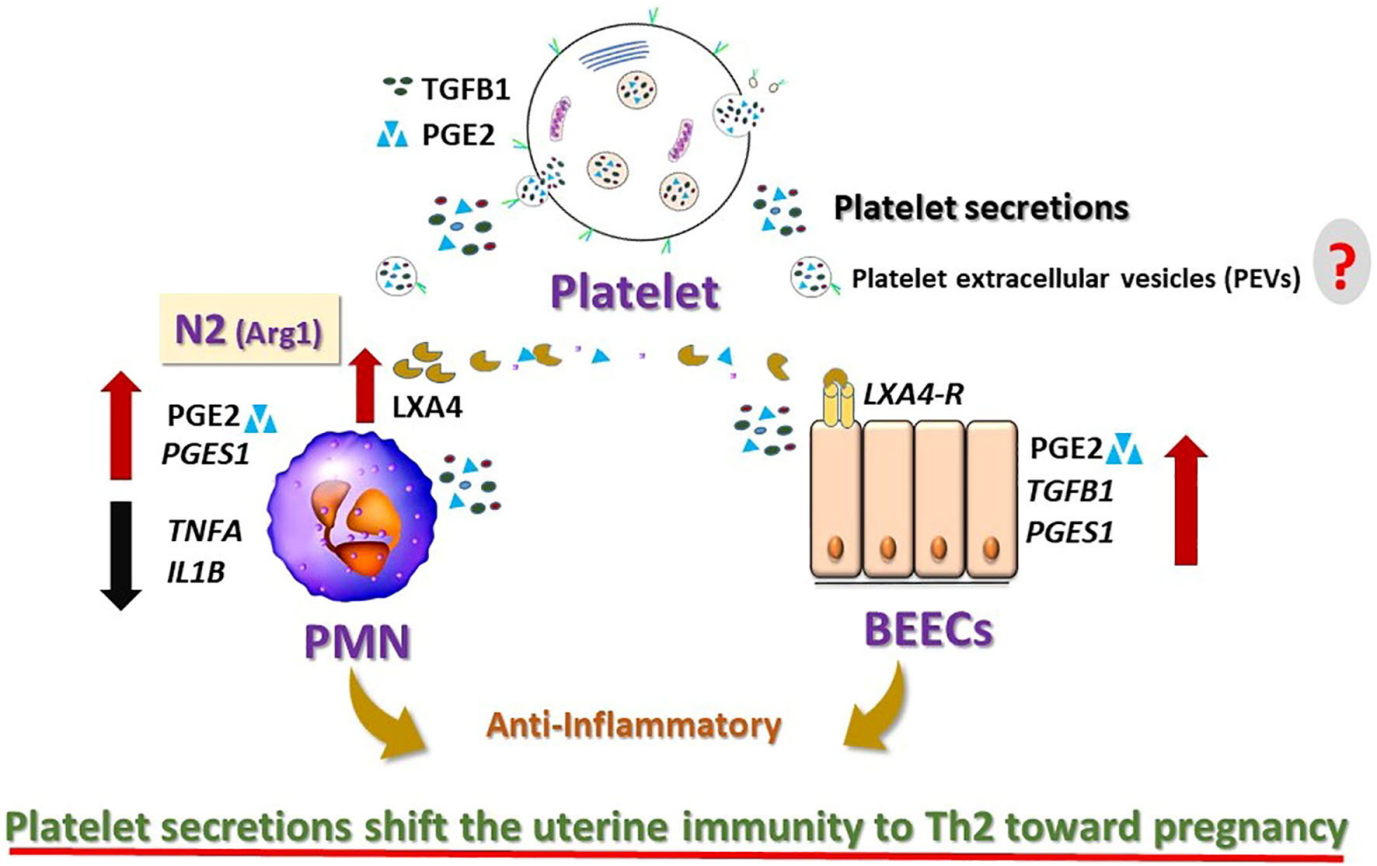
Schematic diagram summarizing the findings and the working hypothesis of the study. Platelet secretions induced anti-inflammatory effects on the bovine neutrophils (PMNs) and endometrial epithelial cells (BEECs). Platelet secretions shift the PMNs toward anti-inflammatory. They trigger the production of Lipoxin A4 (LXA4; anti-inflammatory mediator) from N2-PMNs and upregulate the expression of LXA4-receptor (LXA4-R) in BEECs. LXA4 produced by N2 neutrophils may support the anti-inflammatory response in the bovine uterus by interaction with LXA4-R. Platelets are likely to modulate the innate immunity of the bovine uterus toward anti-inflammatory response and may support the early pregnancy in cattle. However, the exact mechanisms by which the platelets’ components [i.e. platelet extracellular vesicles (PEVs)] in the bovine uterus remain unknown (?).

Overall, the present data provide the first clear evidence that platelets, *via* their secretions, communicate with PMNs and endometrial epithelial cells, to induce anti-inflammatory response in cattle. Such a novel crosstalk could be the part of amplification system in the initial phase of immune network for driving uterine immunity toward anti-inflammatory (Th2) condition for establishing pregnancy in cattle (**Figure 7**). Thus, platelets and/or their derivatives could be used as a potential biological material to modulate the uterine environment and improve fertility. The present findings require further expansion of sample sizes, clarification of the platelet components and *in vivo* validation to confirm their physiological relevance within the bovine uterine environment, to achieve potential clinical applications in the field.

## Data Availability

The original contributions presented in the study are included in the article/supplementary material. Further inquiries can be directed to the corresponding author.
